# Caste development and reproduction: a genome-wide analysis of hallmarks of insect eusociality

**DOI:** 10.1111/j.1365-2583.2006.00696.x

**Published:** 2006-10-01

**Authors:** A S Cristino, F M F Nunes, C H Lobo, M M G Bitondi, Z L P Simões, L Da Fontoura Costa, H M G Lattorff, R F A Moritz, J D Evans, K Hartfelder

**Affiliations:** * Instituto de Matemática e Estatística, Universidade de São Paulo, São Paulo Brazil; † Departamento de Genética, Faculdade de Medicina de Ribeirão Preto, Universidade de São Paulo, Ribeirão Preto Brazil; ‡ Departamento de Biologia Celular e Molecular e Bioagentes Patogênicos, Faculdade de Medicina de Ribeirão Preto, Universidade de São Paulo, Ribeirão Preto Brazil; § Departamento de Biologia, Faculdade de Filosofia, Ciências e Letras de Ribeirão Preto, Universidade de São Paulo, Ribeirão Preto Brazil; ¶ Instituto de Física de São Carlos, Universidade de São Paulo, São Carlos Brazil; ** Institut für Biologie, Molekulare Ökologie, Martin-Luther-Universität Halle-Wittenberg, Halle (Saale) Germany; †† Bee Research Laboratory, USDA-ARS, BARC-E, Beltsville, MD USA

**Keywords:** caste development, oogenesis, meiosis, UCR motifs, AlignACE

## Abstract

The honey bee queen and worker castes are a model system for developmental plasticity. We used established expressed sequence tag information for a Gene Ontology based annotation of genes that are differentially expressed during caste development. Metabolic regulation emerged as a major theme, with a caste-specific difference in the expression of oxidoreductases vs. hydrolases. Motif searches in upstream regions revealed group-specific motifs, providing an entry point to *cis*-regulatory network studies on caste genes. For genes putatively involved in reproduction, meiosis-associated factors came out as highly conserved, whereas some determinants of embryonic axes either do not have clear orthologs (*bag of marbles*, *gurken*, *torso*), or appear to be lacking (*trunk*) in the bee genome. Our results are the outcome of a first genome-based initiative to provide an annotated framework for trends in gene regulation during female caste differentiation (representing developmental plasticity) and reproduction.

## Introduction

The evolution of social organization in the Hymenoptera is intricately linked to the division of reproductive activities between highly fertile queens and functionally sterile workers (Wilson, 1971). Ontogenetically, these alternative phenotypes primarily reflect the differential feeding of larvae, a mechanism that is especially pronounced in the honey bee, *Apis mellifera*. Queen-destined larvae are fed large amounts of royal jelly during the entire larval feeding phase, whereas larvae destined to become workers receive an altered diet during the last larval instars (Haydak, 1970). This differential feeding program, in turn, acts on the endocrine system where it generates caste-specific signatures in juvenile hormone (JH) and ecdysteroid titres (Hartfelder & Engels, 1998; Rachinsky *et al*., 1990). These metamorphic hormones are part of the endocrine programme that drives morphogenesis into either of the two alternative pathways.

The major differences between an adult honey bee queen and a worker reside in the reproductive system. A queen usually has close to 200 ovarioles per ovary and is capable of producing several hundred eggs per day. Workers in contrast have between two and 12 ovarioles per ovary (Snodgrass, 1956), which do not show signs of ongoing oogenesis as long as the queen is present. If the queen is lost, a number of workers can activate their ovaries and produce haploid eggs that will develop into drones (Kropácová & Haslbachová, 1971; Page & Erickson, 1988; Moritz *et al*., 1996).

In order to come to an understanding of the molecular nature and the signal transduction pathways underlying these developmental and ovary activation signals, differential gene expression profiling in honey bee caste development was initiated in the late nineties. The main body of currently available data resulted from a cDNA library generated by suppression subtractive hybridization (SSH) that contrasted queen and worker larvae (Evans & Wheeler, 1999). Subsequent macroarray analyses (Evans & Wheeler, 2000) revealed a clustering of these expressed sequence tags (ESTs) into three distinct groups: genes overexpressed in young (bipotent) larvae, genes overexpressed in fifth-instar queen larvae, and genes overexpressed in fifth-instar worker larvae. A second study focusing on oxidative metabolism identified a set of differentially expressed mitochondrial genes (Corona *et al*., 1999). The third approach was a DDRT-PCR screen for hormone responsive genes to investigate the mode of action of ecdysteroids in the differentiation of the larval ovary (Hepperle & Hartfelder, 2001). Many of these EST sets could not be properly annotated at that time, either because of a limited number of fully sequenced insect genomes, or because the libraries contained large numbers of transcripts in 3′-gene regions, including poorly conserved untranslated regions (UTRs). The draft assembly for the honey bee genome (Honey Bee Genome Sequencing Consortium, 2006) now permits a much more reliable annotation of this unique set of experimentally validated genes.

Reproductive activity of honey bees is determined in a two-step process. The basic differences in reproductive capacity between queen and workers manifest themselves during larval development by a wave of programmed cell death that leads to the destruction of over 95% of the ovariole primordia in the larval ovary of workers (Schmidt-Capella & Hartfelder, 1998). In the adult life cycle of each caste, the co-ordinated flux of egg production through previtellogenic and vitellogenic growth will require the activity of other sets of genes. Some of these act as determinants of the major egg and also embryonic axes. As the fruit fly is the most well developed insect model for axis determination (St Johnston & Nüsslein-Volhard, 1992), and maternal factors have not yet been functionally characterized in the honey bee, searching the genome assembly (Honey Bee Genome Sequencing Consortium, 2006) provides the first major opportunity to explore putative patterning networks in honey bees.

The vitellogenic growth phase of the honey bee oocyte has long been the centre of attention as a means of describing differential fertility of the female castes (Engels, 1974). The synthesis of large amounts of vitellogenin by the queen fat body is intimately related to her high reproductive rate. The equally high vitellogenin titres in haemolymph of nonreproducing young worker bees, however, have been an enigma as their ovaries are inactive in the presence of the queen. Vitellogenin expression has apparently become uncoupled from oocyte growth during the evolution of the sterile worker caste and has acquired secondary functions. It became involved in the production of royal jelly (Amdam *et al*., 2003) and in the regulation of worker lifespan (Amdam *et al*., 2004) through an inhibitory effect on the endocrine system (Guidugli *et al*., 2005). Along with such unique life-history traits related to socially organized reproduction, honey bees also promise to answer new questions involving meiosis, as the honey bee genome exhibits recombination rates that exceed those of all other higher organisms (Hunt & Page, 1995; Solignac *et al*., 2004) and as honey bee males, being haploid, forego meiosis I in producing gametes.

The honey bee genome sequence database (Honey Bee Genome Sequencing Consortium, 2006) has become an extremely valuable resource not only for comparative genomics, but also for functional genomics. One of the oldest, and for evolutionary biologists most challenging question in social insect biology is the development of a reproductive and a nonreproductive caste (Darwin, 1859). Apart from its implications on evolutionary theory in terms of kin selection (Hamilton, 1964), this is essentially a question of how developmental pathways diverge to shape distinct phenotypes, and how oogenesis is regulated to achieve levels of extremely high (queen) and extremely low (worker) fertility.

The annotation of genes related to caste development and differential reproduction in the honey bee has implications well beyond this species. It represents the first genome-wide annotation of a molecular architecture behind reproductive division of labour. In the light of current discussions on the importance of alternative phenotypes in the evolution of novelties (West-Eberhard, 2003) the honey bee genome information is certainly one of the most valuable resources. In the present manuscript we delineate a strategy on how to transcend from a straightforward gene annotation approach to functional studies based on motif analysis of upstream regulatory regions.

## Results and discussion

### From caste to *BLAST*: differentially expressed genes in caste development

The full list of genes that are overexpressed in fifth-instar queen or worker larvae, is made available online in the Supplementary material (Table 1S). This list includes scaffold number, corresponding EST number(s), GLEAN3-predicted protein sequence, similarity and identity indices to corresponding *Drosophila melanogaster* orthologs, as well as protein domain information (Pfam).

A general result was that a relatively large subset of genes (nine of 34) overexpressed in honey bee queen larvae is represented by putative *Drosophila* orthologs for which no Gene Ontology (GO) term for Biological Process is indicated in Flybase. In contrast, all worker genes correspond to functionally relatively well-defined *Drosophila* genes. Even when taking into consideration the conceptual limits in attributing GO terms on biological process from *Drosophila* orthologs to honey bee genes, this finding could have a bearing on basic questions in socioevolution, namely, which caste is the novelty, the queen or the worker(s)? Phrased in other terms, the genome sequence information now permits to address at a molecular level questions that are fundamental to understand the role of (and evolutionary trends in) ontogenetic processes that structure insect societies, especially in hymenopterans. Such basic questions are (1) how many degrees of freedom (or release from constraints) may actually have been gained from splitting the functions normally performed by a solitary ancestral hymenopteran female into two or more castes, and (2) how was this release from constraints integrated into postembryonic differentiation processes to generate truly alternative phenotypes. A second observation of potential interest to functional genomics was that a relatively large subset of the caste-related genes maps to chromosome 2 (seven of 51 unique sequences).

Most genes in the caste gene list are represented by one or two EST hits, except for a predicted *hexamerin 70b* gene (GB10869-PA). This gene was evidenced by 10 ESTs, one in a 5′-located exon and nine in the 3′ region (five ESTs comprising parts of exon 7 and parts of the 3′-UTR, the other four ESTs landing in exons 6 and 7). The macroarray data (Evans & Wheeler, 2000) established this gene as overexpressed in the worker caste. Hexamerins are an important class of storage proteins that show interesting expression patterns related to caste and reproduction in many social insects (Martinez *et al*., 2001; Hunt *et al*., 2003; Zhou *et al*., 2006a,b). A cDNA encoding the honey bee Hexamerin 70b subunit has recently been cloned and sequenced (Cunha *et al*., 2005), and hormone manipulation experiments showed that the abundance of *hexamerin 70b* transcripts in larval development is positively correlated with high levels of JH and ecdysteroids. This could actually reflect a regulatory feedback function in JH titre regulation, as exemplified in the termite *Reticulitermes flavipes*, where the Hex1/Hex2 ratio controls JH availability for caste-specifically differentiating tissues (Zhou *et al*., 2006b).

**Figure 1:**
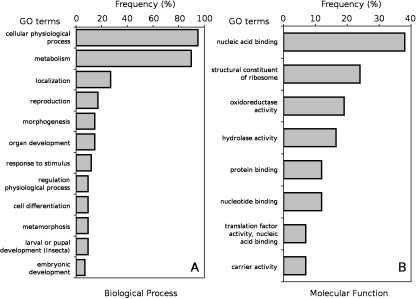
Dominant gene ontology terms for (A) Biological Process and (B) Molecular Function in honey bee genes with an experimentally validated caste-specific expression pattern during the last larval instar. The graph was generated by a FatiGO analysis set at level 3. Frequencies indicate the appearance of GO terms in the total set of queen and worker differentially expressed genes.

**Figure 2:**
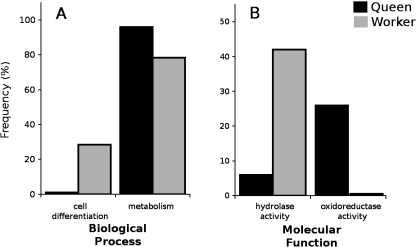
Gene Ontology categories with caste-specific expression patterns for Biological Process (A). Genes classified as part of cell differentiation processes are significantly overexpressed in workers, whereas genes related to metabolism are overexpressed in queen larvae. In the Molecular Function categories (B) we observed an apparent split indicating differential enzyme preferences in queens (overexpress oxidoreductases) and in workers (overexpress hydrolases). The graph was generated by a FatiGO analysis set at level 3. Frequencies indicate the appearance of GO terms in the queen (black bars) and worker differentially expressed genes (grey bars).

Within the honey bee caste genes for which GO information was imported and deduced from their *Drosophila* orthologs we noted a predominance of terms clustering as ‘cellular physiological process’ (95%; GO:0050875) and ‘metabolism’ (90%; GO:0008152) in the ‘Biological Process’ (GO:0008150) category (Fig. 1A). GO-statistics differences between queens and workers became apparent in terms clustering as ‘cell differentiation’ (0% for queen and 28.5% for workers; GO:0030154) and ‘metabolism’ (96% for queen and 78.5% for worker; GO:0008152) in the ‘Biological Process’ (GO:0008150) (Fig. 2A).

With respect to ‘Molecular Function’ (GO:0003674), most terms were related to mRNA translation (‘nucleic acid binding’ (38%; GO:0003676), ‘structural constituent of ribosome’ (24%; GO:0003735), ‘protein binding’ (12%; GO:0005515), ‘nucleotide binding’ (12%; GO:0000166), ‘translation factor activity, nucleic acid binding’ (7%; GO:0008135). Further important terms were ‘oxidoreductase activity’ (19%; GO:0016491) and ‘hydrolase activity’ (16.5%; GO:0016787) (Fig. 1B). For these latter two terms we noted potentially interesting differences related to caste, with ‘hydrolase activity’ being overrepresented by worker transcribed genes, whereas ‘oxidoreductase activity’ was exclusively represented by queen genes (Fig. 2B). Even though these GO assignments on Molecular Function are based on evidence from *D. melanogaster*, without experimental evidence for *Apis mellifera*, the corresponding genes are well conserved in sequence and show the relevant protein domains (Supplementary material, Table 1S*)*, and thus, are indicative of functional trends.

In general terms, the caste-specific separation into metabolic pathway preferences, oxidoreductases vs. hydrolases, may reflect the switch in diet that a worker larva experiences during the fourth and fifth larval instar. This represents a switch from a protein/lipid-rich diet to a more carbohydrate-rich diet (Haydak, 1970), and this switch apparently is accompanied by an increase in the expression of genes coding for proteins with hydrolase activity. Similar switches in gene expression patterns have recently been reported for *D. melanogaster* in an experiment where larvae were shifted from a cornmeal diet to a banana diet (Carsten *et al*., 2005), resulting in the up- or down-regulation of 55 genes of a test population of 6000. Among these are five genes with dehydrogenase/oxidoreductase activity. These parallels in dietary switch responses are indicative of conserved coregulated gene networks. An open question is, of course, how these can be co-opted to generate different phenotypes, such as the castes of social insects. In this respect, social insects clearly go a big step beyond the simple metabolic switch response seen in *Drosophila.* They have apparently incorporated divergent metabolic regulation into a network architecture consistent with morphogenetic differentiation. This required that metabolic regulation became integrated through the endocrine system with developmental patterning processes.

The importance of metabolic regulation on caste development has also come to light in a recent Representational Difference Analysis (RDA) study on caste development in the highly eusocial stingless bee *Melipona quadrifasciata* (Judice *et al*., 2006). This is particularly interesting because in this genus, caste development is thought to be based on a genetic predisposition (Kerr, 1950). Metabolic regulation may, thus, be a *sine qua non* for caste development, and caste-specific metabolic pathways may be set in motion rather independently of the nature of the initial switch (nutritional or genetic). The question of how this metabolic switch may integrate with the resultant endocrine signature characteristic for each caste is still a widely open field, but, recent studies in *Drosophila* showing an interaction between ecdysone and insulin signalling in the determination of body size (Colombani *et al*., 2005; Mirth *et al*., 2005) may provide a lead.

This is also the point to reflect on how justified it is to heuristically rely on *Drosophila* orthologs and to use their GO attributes in a developmental context (caste differentiation) that has no parallel in *Drosophila.* A recent gene expression profiling study in the ant *Camponotus festinatus* employing a microarray set-up of 384 clones showed significantly different expression levels for larval vs. adult ants in 91 genes (21 confirmed by qRT–PCR), including an *Apis hexamerin 70b* ortholog (Goodisman *et al*., 2005). When comparing the temporal expression patterns of these ant genes with expression profiles for their respective *Drosophila* orthologs (Arbeitsman *et al*., 2002) relatively little accord was noted for the two species, leading to the suggestion that these genes may have taken on distinct functions due to the long divergence time between dipterans and hymenopterans (Goodisman *et al*., 2005). Differences aside, these examples show that in practically all studies on large-scale functional considerations in gene expression, we are strongly wedded with *Drosophila*, and even though functional divergence in orthologs may have occurred, there is little experimental gene-by-gene evidence available for any of the major insect orders outside of Diptera.

Functional studies are clearly profiting from the now available honey bee genome sequence, as evident from the increasing number of RNAi experiments in honeybees (see citations in Honey Bee Genome Sequencing Consortium, 2006). This is still a small number compared with the large-scale RNAi assays established for *Drosophila* (Boutros *et al*., 2004), but the development of cell culture approaches in the honey bee (Bergem *et al*., 2006) represents a step in this direction.

Alternatively, regulatory functional associations between genes and their integration into networks can be inferred from the presence of response elements in upstream control regions. In our analysis of differentially expressed genes in queen-worker development we took a bioinformatics approach for a first look into the molecular architecture of a developmental polyphenism.

### Motif search in upstream regions of differentially expressed genes

The genes related to caste development are among the first honey bee genes for which experimentally validated expression data were generated (Corona *et al*., 1999; Evans & Wheeler, 1999, 2000; Hepperle & Hartfelder, 2001; Guidugli *et al*., 2004). Certainly, these 51 genes do not comprise all the genes involved in caste development but they are expected be prominent players as they were the ones that stood out in the SSH and DDRT-PCR approaches. The 51 caste genes do not represent gene families but rather fall into many very different molecular function categories. This made us ask whether the observed overexpression pattern of different genes in either queen or worker larvae may be associated with the occurrence of specific regulatory motifs in the upstream control regions (UCR) of these genes.

Three different algorithms, AlignACE (Roth *et al*., 1998), MEME (Bailey & Elkan, 1995) and MDscan (Liu *et al*., 2002) were used to construct a pipeline for detecting overrepresented motifs in the two unaligned sets of UCR sequences for the caste-specifically expressed genes. This pipeline was run on a ‘top-10’ set of 12 genes (six for each caste), which showed the most pronounced caste differences in expression (Evans & Wheeler, 2000) and also on a randomly selected set of UCRs (background control). We calculated four different metrics for each motif: MAP score (Roth *et al*., 1998), a group-specificity score (Church score) (Hughes *et al*., 2000), and a ROC AUC and MNCP metric (Clarke & Granek, 2003). A first set of filters was used to detect motifs with a potential for regulatory functions (MAP score ≥ 5; ROC AUC ≥ 0.7). This resulted in 46 motifs out of 123 total UCR motifs found in the queen UCR set and in 71 motifs out of 261 total found in the worker UCR set (Supplementary material, Table 2S*)*.

A parametric statistical test (manova; *P* = 0.0001; Wilks’ = 0.78; *F* = 7.2) and a nonparametric statistical test (Kolmogorov–Smirnov; Table 1) on ROC AUC and MNCP indices showed that these two sets of filtered motifs are significantly different from a randomly selected set of motifs. The rank-order metrics, ROC AUC and MNCP, have previously been used to compare the association of short regulatory sequence features with gene expression data (microarray analyses on coregulated genes) and they have been useful in flagging false positives erroneously included in ‘top-10’ sets of differentially expressed genes (Clarke & Granek, 2003).

**Table 1:** Kolmogorov–Smirnov analysis of ROC AUC and MNCP metric for statistical significance of putative regulatory motifs in upstream control regions of genes with queen or worker-specific expression patterns. These motifs were contrasted with a random set of motifs detected in a random set of UCRs of GLEAN3-predicted honey bee genes.

Group pairs	ROC AUC	MNCP
Random × (Queen + Worker)	*P* > 0.1	*P* < 0.001
Random × Queen	*P* > 0.1	*P* < 0.005
Random × Worker	*P* > 0.1	*P* < 0.001
Queen × Worker	*P* < 0.1	*P* > 0.1

**Figure 3:**
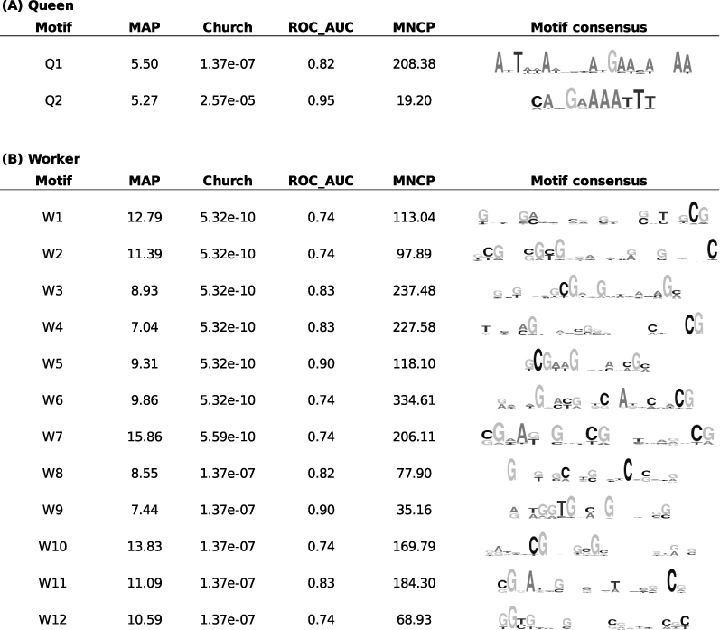
Putative regulatory motifs and their consensus sequences in UCRs of queen and worker overexpressed genes. Scores for MAP, Church, ROC AUC and MNCP metrics indicate degree of group specificity and significance level.

To select highly specific motifs found in each data set we used the group-specificity score (Church score ≤ 1e^−05^; Hughes *et al*., 2000) to identify the most likely motifs involved in decision making for pathways leading to queen (two motifs, Fig. 3A) or to worker development (12 motifs with Church score ≤ 1e^−07^, Fig. 3B). As the SSH and DDRT-PCR approaches on caste development can be expected to retrieve only a subpopulation of such genes, these motifs represent only a partial scenario of the transcriptional regulatory network underlying caste development. The motifs can now be used to screen other GLEAN3-predicted genes to integrate a candidate list of putatively coregulated genes in caste development that can be submitted to further experimental validation.

Each motif found in UCRs of queen (46) and worker (71) overexpressed genes was compared with the entire set of *D. melanogaster cis*-regulatory motifs contained in the TRANSFAC database (version 4.0; Wingender *et al*., 2000). Only alignments passing 80% identity for each position-specific site matrix (PSSM) were considered as significant matches. Whereas none of the most specific motifs for each caste showed similarity to any of the *D. melanogaster* motifs, some of the more ubiquitous ones did resemble binding sites of transcription factors, such as *Antennapedia, Ultrabithorax*, *zerknüllt*, *even skipped*, *trithorax-like*, *tailless*, *paired*, *fushi tarazu* and *Adh transcription factor 1* (Supplementary material, Table 2S).

**Figure 4:**
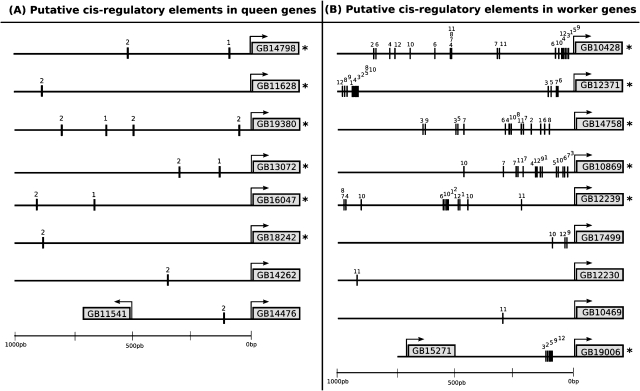
Map of the group-specific motifs found in queen and worker UCRs of caste-specifically expressed genes. The coding region is represented by the GLEAN3 prediction number (assembly 4.0) with arrows indicating the translation start site. Asterisks mark UCRs of the ‘top10’ set used to find the over-represented motifs.

When we plotted the positions of the two queen and the 12 worker motifs in the UCRs of the caste-specifically expressed genes (Fig. 4) an interesting pattern emerged for the worker-specific motifs. Some of the worker motifs appeared to be clustered and occurring in tandem; furthermore, they were positioned relatively close to the predicted translation start sites in some of the genes that are overexpressed during worker development (annotation results of these genes are listed in Supplementary material, Table 1S). A position close to the predicted translation start sites is generally taken as a sign of strong regulatory effect (Davidson, 2001).

As caste development is highly dependent on changes in haemolymph titres of JH and ecdysteroids we also screened the UCRs of the differentially expressed genes for putative nuclear receptor binding sites. Regulatory elements involved in the JH response are not well understood yet, so any prediction in this direction would be elusive (Wheeler & Nijhout, 2003). Functional ecdysone response elements (EcRE) have, however, been identified and it is now well established that the EcR/USP complex binds to direct or inverted (palindromic) repeats (Riddihough & Pelham, 1987; Antoniewski *et al*., 1995; Perera *et al*., 2005). A PSSM search (Wassermann & Sandelin, 2004) based on a canonical representation (rGkTCAaTGamcy) (Perera *et al*., 2005) did not reveal any putative EcRE motif in the UCRs of the 51 caste-differentially expressed genes. However, this does not rule out that these genes respond to changes in JH and/or ecdysteroid titres as these hormones require EcR/USP binding primarily in the expression of early genes, but not necessarily for the late response genes (Li & White, 2003; Sullivan & Thummel, 2003).

In conclusion, the predictions from such a combined strategy that searches for group-specific and for conserved regulatory motifs in GLEAN3 predicted honey bee genes represents a major transition from nonhypothesis-driven high-throughput screens to hypothesis-driven searches for context-dependent gene expression in honey bees. Such directed search results can serve as a platform for experimental analyses of genome-wide integration in hormonal control of caste development in bees. In addition, this study exemplifies how existent algorithms for detecting shared regulatory motifs can be joined into a toolkit for predicting coregulated gene expression patterns in honey bees. These methods have been shown to be robust and are gaining acceptance for use in functional and comparative genomics (Liu *et al*., 2004; Pritsker *et al*., 2004; Zhu *et al*., 2005).

### Oogenesis and reproduction

As caste development sets the stage for reproductive division of labour, genes involved in reproductive processes are strong candidates for functional analyses. In the present study we performed BLAST searches to identify honey bee orthologs for a list of 32 fly genes with the GO attribute ‘oogenesis’ and for four genes specifically related to ‘vitellogenesis’. The list for fly genes involved in nuclear events in germ cells consisted of 20 genes for ‘female meiosis’, 12 genes for ‘recombination’ and 21 genes under the heading ‘chromosome segregation including segregation distortion’ (Supplementary material, Table 3S). In some cases, these GO attributes for fly genes, of course overlapped.

BLASTN and BLASTX searches for these fly genes against the honey bee genome assembly 3.0 and the GLEAN3 Official Set (aa) retrieved statistically well supported putative bee orthologs for most of these candidates. For the genes involved in meiosis, recombination and chromosome segregation this finding, although not unexpected, is of interest as meiosis in the haploid honey bee drone is strongly modified when compared with a normal diploid meiosis. The first meiosis is initiated but the nucleus remains undivided and only the superfluous centrioles are eliminated as cytoplasmatic buds (Hoage & Kessel, 1968). An interesting gene, thelytoky (*th*), has recently been mapped in this context (Lattorff *et al*., 2005). It prevents almost completely meiotic recombination in the automixis of laying workers of the Cape honey bee. As an indication of the interplay between meiosis and later development, this locus also appears to be an integral part of various gene cascades involved in caste determination (Lattorff *et al*., 2006).

The fly genes retrieved in the GO searches for ‘oogenesis’ represent a much larger range of Molecular Function categories, such as transcription factors, proteins regulating translation by RNA binding, RNA helicases, enzymes (ubiquitination, transfer of sugar residues, sulfotransferase), GTPase activity, and several factors binding to cytoskeletal proteins (Supplementary material, Table 3S). This wide range of functional categories is expected as these genes are involved in a series of different steps during oogenesis in the polytrophic meroistic ovary. Oogenesis starts out with the maintenance of germline and somatic stem cell identity in the germline niche in the upper germarium. A key gene involved in this process is *pumilio* (Forbes & Lehmann, 1998), which is represented by a highly conserved bee ortholog, GB10504-PA. The second step is the formation of germ cell cysts, the determination of an oocyte within each cyst, and the survival of these cysts, involving genes such as *benign gonial cell neoplasm* (Lin *et al*., 1994), *encore* (Hawkins *et al*., 1996), *ovo* and *ovarian tumour* (*otu*) (Staab & Steinmann-Zwicky, 1995), all well conserved in the honey bee genome. Interestingly, we could not find a clear bee ortholog for *bag of marbles* (*bam*), which is one of the prime early response genes in the cystoblast differentiation pathway in *Drosophila* (McKearin, 1997).

The third step comprises previtellogenic growth of the follicle, and during these stages a number of maternal factors are deposited and anchored either within the oocyte or in the perivitelline space that define the egg and the future embryonic axes (for review see, St Johnston & Nüsslein-Volhard, (1992). In the list of *Drosophila* genes involved in early steps of axis determination a couple of surprises came up in the search for honey bee orthologs. A big surprise was that we could not find a *gurken* ortholog in the bee, even though this gene sets up both the anterior–posterior and dorsal–ventral axes in the *Drosophila* egg (González-Reyes *et al*., 1995), whereas downstream components of the Gurken signalling cascade appear to be preserved in the bee genome (Honey Bee Genome Sequencing Consortium, 2006). Similar apparent gaps in constituents of patterning cascades were noted for the terminal regions of the embryo, such as a lack of a *torso* ortholog, whereas its ligand, *torso-like*, is represented by a well conserved ortholog in the bee (GB18663-PA).

With respect to genes involved in the final processes of oogenesis we primarily looked at genes that play a part during vitellogenesis. There are four genes of interest in this class, the primary one coding for the yolk protein precursor vitellogenin. This gene has already been sequenced for the honey bee (Piulachs *et al*., 2003) and, as expected, it is much more related to vitellogenins of other insects and even vertebrates than to the *Drosophila* yolk proteins, which apparently are derived from lipases (Hagedorn *et al*., 1998). The second gene of interest is the bee ortholog to *yolkless*, as this (GB16571-PA) could represent a putative vitellogenin receptor. The other two *Drosophila* genes with clear orthologs in the bee are CG18641 and CG12139, which code for a lipase and an LDL receptor, respectively.

### General conclusions

The current analysis made use of previous experimental analyses on differential transcription during caste development of honey bee larvae. In the annotation of these genes, which includes references to Gene Ontology terms associated with their respective *Drosophila* orthologs, two major configurations emerged. First of all, worker genes were better defined in terms of GO attributes, compared with the relatively large number of queen genes that had no GO terms associated to their respective *Drosophila* orthologs. Even when taking into consideration the conceptual limits in attributing GO terms on molecular function and biological process from *Drosophila* orthologs to honey bee genes, this finding could have a bearing on general basic questions in socioevolution, namely, which caste is more divergent from a nonsocial reproductive female bee prototype or reproductive ground plan, the queen or the worker? Less speculative is the second major conclusion coming out of the GO analysis for Molecular Function, showing and confirming (Eder *et al*., 1983; Corona *et al*., 1999) the important role of metabolic regulation in caste development. This facet is demonstrated especially clearly in the caste-specific expression of oxidoreductases (queen) vs. hydrolases (workers).

The honey bee genome information provided not only a much improved annotation platform for caste-specifically expressed ESTs, but, even more so, opens the possibility to explore putative regulatory features of the honey bee genome. In the current study we employed modified Gibbs sampling and expectation-maximization algorithms (AlignACE, MDScan, MEME) to detect group-specific motifs in gene regions up to 1000 bp upstream of translation start sites. We detected 14 motifs that were significantly overrepresented in the caste genes, when compared with corresponding motifs found in a random set of GLEAN3-predicted honey bee genes. The localization of such motifs in UCRs of worker-overexpressed genes revealed a clustering of such motifs close to the predicted basal promotor regions suggesting strong regulatory effects. Such search strategies and the detected motifs can provide the lead to reveal and unravel *cis*-regulatory networks for and within specific contexts of honey bee biology.

Caste polyphenism in social insects makes a strong case for the emergence of novelties at a microevolutionary level (West-Eberhard, 2003). Without the pretension to discuss exhaustively the mechanisms underlying this surge of developmental plasticity, two major themes become apparent in this and other studies. Regulatory change has been demonstrated in the shut-down of wing disc patterning cascades in ants (Abouheif & Wray, 2002) and is certainly also implicit in observed temporal changes in gene expression during postembryonic development of ants and bumble bees (Goodisman *et al*., 2005; Pereboom *et al*., 2005). Such change would be expected to involve *cis*-regulatory elements, that is, change in transcription factor binding sites in UCRs, as approached in this study, and also evolutionary change in response thresholds to circulating morphogenetic hormones (for review see, Hartfelder & Emlen, 2005). The second and quite unexpected theme is the acquisition of new systemic functions by evolutionary rather old proteins, such as vitellogenin and hexamerins. These apparently unspectacular proteins have evolved into key players for caste evolution and reproductive division of labour via novel regulatory connectivity with JH (Amdam *et al*., 2004; Guidugli *et al*., 2005; Zhou *et al*., 2006a,b).

## Experimental procedures

### Selection and annotation of ESTs representing differentially expressed genes in honey bee caste development

The starting point were 164 entries (mainly 3′-ESTs) in GenBank (BG101532–BG101697) from an SSH library (Evans & Wheeler, 1999; Evans & Wheeler, 2000). When validated by macroarray analyses, a clustering into three major classes became apparent: (I) genes overexpressed in young larvae; (II) genes overexpressed in last instar queen larvae; and (III) genes overexpressed in last instar worker larvae. For this study we excluded the class I ESTs because their expression is not caste-specific, but rather represents expression differences between young (still bipotent) and older larvae. To the class II queen ESTs (82) we added one complete cDNA entry (AY601642) from a DDRT-PCR screen (Corona *et al*., 1999), and to the class III set of worker ESTs (40) we added seven GenBank dbEST entries (BG149167–BG149173) from a DDRT-PCR screen on ovary development (Hepperle & Hartfelder, 2001).

The EST sequences were submitted to BLASTN searches (parameters: -G 2 -E 3 -W 15 -F ‘m D’ -U -e 1e-20) against genome sequence assembly Amel_v3.0 to retrieve matches in linked or unlinked genomic contigs and to exclude no-matches (seven ESTs in queen). ESTs that aligned within the same scaffold were checked for clustering and overlap. This clustering also served to exclude genes that were represented by non-overlapping ESTs from both castes. This procedure generated a set of 51 unique putative gene sequences overexpressed in either queen (34) or in worker larvae (17). These 51 nonredundant sequences were submitted to BLASTX searches against the Official Set of GLEAN3-predicted protein sequences (cut-off value at 1e^−20^). For ESTs with no significant protein sequence matches, the genomic regions adjacent to the mapped EST were searched to find neighbouring ORFs, especially those nearest to putative 3′ UTRs of predicted proteins, as the EST libraries have a bias in this direction.

Official Set protein sequences were aligned against Amel_v3.0 sequence assembly using TBLASTN to map protein to genome and subsequently, they were aligned using BLASTP against the GenBank nonredundant (nr) and the Flybase protein sequence databases. The manual features annotation procedure of the Artemis 7.0 program (Rutherford *et al*., 2000) was used to map ORFs, putative splice sites of exons and ESTs to genome coordinates. The final annotation file was generated with a Python script in GFF format (http://www.sanger.ac.uk/Software/formats/GFF/).

Honey bee sequences annotated as orthologs to *D. melanogaster* genes were putatively assigned the GO terms listed in the respective Flybase entry. In addition, the definition of new GO terms (Biological Process ontology) related to caste development and polyphenism (GO:0048651 and GO:0048650, respectively) was co-ordinated with the Gene Ontology Consortium (Ashburner *et al*., 2000). The FatiGO web tool (Al-Sharour *et al*., 2004) was used to cluster GO terms (level 3 setting) for Biological Process and Molecular Function.

For the detection of conserved domains, the 51 protein sequences were screened against the Pfam database (http://www.sanger.ac.uk/Software/Pfam/) using the HMMER platform (current release 2.3.2, http://hmmer.wustl.edu/), with a cut-off value set at 1e^−10^.

### Annotation of oogenesis and reproduction genes

In order to identify putative honey bee orthologs to *D. melanogaster* genes we searched the following GO terms in Flybase: ‘oogenesis’ (GO:0009993), ‘vitellogenesis’ (GO:0007296), ‘female meiosis’ (GO:0007143), ‘DNA recombination’ (GO:0006310), and ‘chromosome segregation’ (GO:0007059). Genes related to segregation distortion were searched for in Flybase in phenotypic descriptions and mutant effects of *D. melanogaster* genes as this phenomenon is not represented by a GO term. Hence this group may be more heterogeneous than the others. From this list we removed genes of pleiotropic function (multifaceted GO entries in Biological Process) and genes that lacked defined transcripts in the *Drosophila* genome database.

For the GO terms ‘oogenesis’ and ‘vitellogenesis’ we performed TBLASTN and BLASTP searches for 42 fruit fly genes against the Amel_v3.0 genome assembly and the GLEAN3-predicted protein sequences (Honey Bee Genome Sequencing Consortium, 2006), respectively. The orthologous *D. melanogaster* gene was characterized by the same procedure as described above (reciprocal best hit). For the GO terms ‘female meiosis’, ‘DNA recombination’, ‘chromosome segregation’ and the non-GO group ‘segregation distortion’, transcripts of *D. melanogaster* were searched against nr databases at NCBI using BLASTP. The obtained sequences were searched against the Amel_v3.0 genome assembly and the GLEAN3-predicted protein sequences using TBLASTN. Homologous sequences (threshold 1e^−10^) were predicted using the BioEdit software (Hall, 1999). ORFs showing significant homology (BLASTP, threshold 1e^−20^) were assembled and used in BLASTP searches against the nr databases at NCBI.

### Motif search in upstream regions in caste-specifically expressed genes

In order to detect overrepresented motifs in the upstream control regions (UCRs) of the two sets of caste-related genes we selected gene subsets based on two criteria: (1) those that had shown the highest caste-specificity in the array analyses (Evans & Wheeler, 2000), and (2) those that had a conserved 5′ region when compared with the *Drosophila* orthologs. These ‘top10’ genes consisted of six queen genes (GB13072, GB11628, GB19380, GB14798, GB16047 and GB18242) and of six worker genes (GB10869, GB12371, GB12239, GB10428, GB19006, GB14758). The motif search was conducted separately on the two sets of UCR sequences using three methods: AlignAce (Roth *et al*., 1998), MEME (Bailey & Elkan, 1995) and MDscan (Liu *et al*., 2002). Default parameters values were used in all searches, except that GC content in intergenic regions was set to 25%, representing the background value established for the honey bee UCR database generated in this study.

The database containing 10,156 UCR sequences was generated by parsing the Official Set annotation file (downloaded in GFF format from http://www.beegenome.hgsc.bcm.tmc.edu/beeftp.html) to extract upstream regions starting from the terminal 5′-genomic coordinate of each predicted CDS. The UCRs were arbitrarily set to a size frame of 1000 nucleotides (Roth *et al*., 1998), but were trimmed whenever another predicted ORF was detected in any of these regions.

The MAP (maximum a priori log likelihood) score, group specificity score (called Church score in this manuscript) (Hughes *et al*., 2000), ROC AUC (area under the curve for a receiver-operator characteristic plot) metric and MNCP (mean normalized conditional probability) metric (Clarke & Granek, 2003) were used to detect motifs that most likely correspond to biologically significant *cis*-regulatory elements. The filters ran on the UCRs of the subsets of queen and worker genes were a MAP score cut-off value of 5.0, followed by a ROC AUC cut-off at 0.7, followed by a group specificity score cut-off at 1e^−05^. The UCR database of all GLEAN3 predicted honey bee genes was used as the background to calculate these metrics.

A parametric test (MANOVA) and a nonparametric test (Kolmogorov–Smirnov) were conducted to identify significance levels for the two sets of filtered motifs found in the UCRs of caste-specifically expressed genes against filtered motifs found in the random UCR set. Random motifs were sampled from a motif database (10 391 motifs) generated by running our script 100 times with a random sets of UCR sequences.

The main criterion for identifying known regulatory motifs among these caste-specific ones was the alignment of the PSSM for each bee motif with the *Drosophila melanogaster* sequences in the TRANSFAC database (release 4.0) (Wingender *et al*., 2000). Only the alignments passing a threshold of 80% identity for each PSSM were considered as significant matches. In addition, we checked for a specific binding motif, the EcR/USP motif (rGkTCAaTGamcy-3′), known to function in the expression of genes responding to morphogenetic hormone titres (Perera *et al*., 2005).

### Operating system and programming tools

An Ubuntu Linux (version Breezy) operating system was used to implement all scripts and pipelines designed for annotation procedures and motif discovery. The Python programming language (http://www.python.org/), Biopython (http://www.biopython.org), and TAMO (Tools for Analysis of Motifs) packages (Gordon *et al*., 2005) were used in program design. Other web applications were built using the Zope application server (http://www.zope.org) hosted at http://zulu.fmrp.usp.br/beelab.

## Supplementary material

Table S1 Annotation results of caste-specifically expressed honey bee genes.

Table S2 Caste-specific motifs in UCRs of honey bee genes.

Table S3 Annotation of honey bee orthologs to Drosophila melanogaster genes involved in Reproduction.

This material is available as part of the online article from http://www.blackwell-synergy.com.
